# Number of apoptotic cells as a prognostic marker in invasive breast cancer

**DOI:** 10.1054/bjoc.1999.0928

**Published:** 2000-01-18

**Authors:** J S de Jong, P J van Diest, J P A Baak

**Affiliations:** Department of Pathology, Free University Hospital Amsterdam, PO Box 7057, Amsterdam, MB NL-1007, The Netherlands

**Keywords:** breast cancer, apoptosis, proliferation, prognostic factor

## Abstract

Apoptosis plays an important role in tumorigenesis. Tumour growth is determined by the rate of cell proliferation and cell death. We counted the number of apoptotic cells in haematoxylin and eosin (H&E)-stained tumour sections in series of 172 grade I and II invasive breast cancers with long-term follow-up. The number of apoptotic cells in ten high-power fields were converted to the number of apoptotic cells per mm^2^to obtain the apoptotic index (AI). The AI showed a positive correlation to the mitotic activity index (MAI) (*P* = 0.0001), histological grade (*P*< 0.0001) and worse tumour differentiation. Patients with high AI showed shorter overall survival than patients with low AI in the total group as well as in the lymph node-positive group. Tumour size, MAI, lymph node status and AI were independent prognostic indicators in multivariate analysis. The AI was shown to be of additional prognostic value to the MAI in the total patients group as well as in the lymph node-positive group. The correlation between the AI and the MAI points to linked mechanisms of apoptosis and proliferation. Since apoptotic cells can be counted with good reproducibility in H&E-stained tumour sections, the AI may be used as an additional prognostic indicator in invasive breast cancer. © 2000 Cancer Research Campaign


*British Journal of Cancer* (2000) **82**(2), 368–373
© 2000 Cancer Research Campaign
Article no. bjoc.1999.0928

### Number of apoptotic cells as a prognostic marker in invasive breast cancer

**JS de Jong, PJ van Diest and JPA Baak**

Department of Pathology, Free University Hospital Amsterdam, PO Box 7057, NL-1007 MB Amsterdam, The Netherlands

**Summary** Apoptosis plays an important role in tumorigenesis. Tumour growth is determined by the rate of cell proliferation and cell death. We counted the number of apoptotic cells in haematoxylin and eosin (H&E)-stained tumour sections in series of 172 grade I and II invasive breast cancers with long-term follow-up. The number of apoptotic cells in ten high-power fields were converted to the number of apoptotic cells per $mm^2$ to obtain the apoptotic index (AI). The AI showed a positive correlation to the mitotic activity index (MAI) ($P = 0.0001$), histological grade ($P < 0.0001$) and worse tumour differentiation. Patients with high AI showed shorter overall survival than patients with low AI in the total group as well as in the lymph node-positive group. Tumour size, MAI, lymph node status and AI were independent prognostic indicators in multivariate analysis. The AI was shown to be of additional prognostic value to the MAI in the total patients group as well as in the lymph node-positive group. The correlation between the AI and the MAI points to linked mechanisms of apoptosis and proliferation. Since apoptotic cells can be counted with good reproducibility in H&E-stained tumour sections, the AI may be used as an additional prognostic indicator in invasive breast cancer. © 2000 Cancer Research Campaign

**Keywords:** breast cancer; apoptosis; proliferation; prognostic factor

In the last 10 years it has become evident that programmed cell death (apoptosis) plays an important role in tumorigenesis. Tumour growth is not only determined by the rate of tumour cell proliferation but rather by the net result of proliferation and cell death. Apoptosis is regulated positively and negatively by a wide range of gene products. bcl-2 was the first protein described that blocks apoptosis. Besides bcl-2, various other related proteins have been described like bcl-x and its alternative splicing forms bcl-$x_L$ and bcl-$x_S$ (Farrow and Brown, 1996; Yang and Korsmeyer, 1996). Bcl-$x_L$ seems to function like bcl-2 and inhibits apoptosis while bcl-$x_S$ seems to promote apoptosis by inhibiting bcl-$x_L$ and bcl-2 function (Minn et al, 1996). Recently, two other cell death promoting proteins have been discovered, namely bak and bax. Bak and bax heterodimerize with bcl-2 and bcl-$x_L$ and antagonize the effects of bcl-2 and bcl-$x_L$ (Yang and Korsmeyer, 1996). Bax can homodimerize and promote cell death. The balance between these proteins by forming bax/bax, bax/bcl-2 or bax/bcl-$x_L$ homo- and heterodimers can be regulated by several factors, including p53 and c-*myc*, which may render the cells more susceptible to apoptosis by lowering the bcl-2/bax ratio (Miyashita et al, 1995). bcl-2, bcl-x, bak and bax are expressed in normal breast glandular epithelium and in a subset of breast carcinomas (Doglioni et al, 1994; Krajewski et al, 1994, 1995; Barbareschi et al, 1996; Bargou et al, 1996; Kapucuoglu et al, 1997; Sierra et al, 1998; Veronese et al, 1998).

Induction of apoptosis by chemotherapeutics is one of the key factors in cancer therapy. The resistance of cells to undergo apoptosis is in part regulated by the above described gene products. The number of apoptotic cells and the expression of bcl-2 have been found to be related in invasive breast cancer (van Slooten et al, 1998). The number of apoptotic cells found in a tumour may therefore reflect the inclination of tumour cells to undergo apoptosis. Various methods have been used to assess the number of apoptotic cells. Flow cytometry is widely used for assessing the percentage of apoptotic cells but has the disadvantages of being a non-morphological method and losing the tumour material after the measurement. Different staining techniques have been used to highlight apoptotic cells. Popular methods are terminal transferase (TdT)-mediated dUTP nick end-labelling (TUNEL) and in situ end labelling (ISEL) which target fragmented DNA for detection of apoptotic cells (Weisman et al, 1993). However, the number of apoptotic cells can also be assessed in haematoxylin and eosin (H&E)-stained tumour sections. Several studies have used this method on a range of different tumour types (Leoncini et al, 1993; Lipponen and Aaltomaa, 1994; Lipponen et al, 1994; Heatley, 1995; Zhang et al, 1999). In a previous study, we showed that apoptotic cells can be counted in H&E-stained sections with good inter- and intraobserver reproducibility in invasive breast cancer; besides, counting in H&E sections needs no additional staining techniques and is relatively fast. In invasive breast cancer, a high number of apoptotic cells has been related to a high proliferation index, poor tumour cell differentiation and an unfavourable prognosis (Lipponen et al, 1994; Berardo et al, 1998; Zhang et al, 1999). As described above, tumour cell behaviour is not only determined by the rate of proliferation, but also by the rate of cell death. A combination of measurements of proliferation and apoptosis could therefore provide a more realistic prediction of tumour behaviour. The aim of this study was to evaluate the prognostic value of the apoptotic index (AI) and its additional prognostic value to other factors in a series of 172 invasive breast cancers with long-term follow-up.

*Received 17 February 1999*
*Revised 26 July 1999*
*Accepted 3 August 1999*

*Correspondence to:* PJ van Diest

### MATERIALS AND METHODS

### Patients

We used a previously described group of 172 patients with stage I and II invasive breast cancer (Jannink et al, 1995), diagnosed between 1971 and 1981 in the Free University Hospital or the Netherlands Cancer Institute, Amsterdam, The Netherlands. All patients were treated with radical or modified radical mastectomy with complete axillary dissection. Postoperative locoregional radiotherapy was given in all lymph node-positive cases, and none of the patients received any form of adjuvant systematic therapy. Median follow-up time was 90 months (range 4–120). Median follow-up time for the surviving patients was 111 months (range 5–120). The mean age of the patients was 57 years (range 26–92). Eighty-six patients were diagnosed with positive lymph nodes and 86 patients showed negative lymph nodes. The median tumour size was 2.5 cm (range 0.2–10.0). Seventy-seven patients had tumours smaller than 2 cm, 72 patients had tumours 2–5 cm in size and 23 patients showed a tumour size larger than 5 cm.

### Specimen preparation

Fresh operation specimens were cut in slices of approximately 0.5 cm and tumour size was measured. The material was fixed in neutral 4% buffered formaldehyde. Representative tumour samples were taken, taking especial care that the periphery of the tumour was sampled and embedded in paraffin. All lymph nodes were identified in the axillary dissection specimens and embedded in paraffin as well. Four-micrometre thick sections were cut from the paraffin blocks and mounted for routine staining with H&E for diagnosis, histologic typing according to the WHO criteria, histologic grading (Elston, 1987), mitoses counting and apoptosis counting.

### Counting of apoptotic cells

In H&E-stained tissue sections, apoptotic cells show retracted and strongly eosinophilic cytoplasm (Schepop et al, 1996). The nuclear DNA condenses initially at the nuclear membrane, later forming clumps, often falling apart into round and homogeneously dark nuclear fragments. Apoptosis concerns individual cells and does not provoke an inflammatory reaction. Figure 1 shows examples of apoptotic cells. Apoptotic cells were counted using a standard light microscope at a × 630 magnification (× 63 objective, field diameter 275 μm) according to a strict protocol that was previously developed (Schepop et al, 1996). In short, the total number of apoptotic cells was counted in ten fields of vision, systematically spread over the most poorly differentiated area in the periphery of the tumour 0.5 × 0.5 cm in size. This procedure was shown to provide good intra- and interobserver reproducibility (Schepop et al, 1996). All apoptotic counts were expressed per $mm^2$.

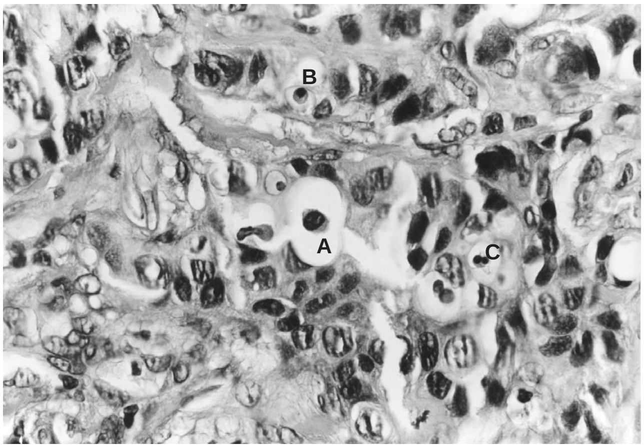

**Figure 1** Examples of apoptotic cells. Early phase apoptosis (A) with condensed chromatin along the nuclear border and retracted cytoplasm. Late phase apoptosis (B) showing homogeneously dark single nuclear remnants and retracted cytoplasm. An apoptotic cell (C) with nuclear fragmentation and retracted cytoplasm

### Mitoses counting

Mitotic figures were counted in the same area as described above in ten consecutive high-power fields at a 400 × magnification using a 40 × objective (field diameter 450 μm), starting at the spot within the measurement field with the highest density of mitotic figures. The total number of mitotic figures counted in these ten fields (1.59 $mm^2$) was taken as the mitotic activity index (MAI) (Baak et al, 1985). The cut off value for the MAI for discriminating between high and low proliferative tumours was ten mitoses per ten high power fields, which was shown to be of strong prognostic value in previous studies by us (Baak et al, 1985; Linden et al, 1987; Uyterlinde et al, 1988; van Diest and Baak, 1992) and others (Clayton, 1991; Lipponen et al, 1991).

### Data analysis

For statistical analysis, grouping was performed using logical classes for the discrete variables, and for the continuous variables the median values were used. To assess correlations, confusion matrices were computed and tested for significance with the $\chi^2$ test. For the continuous variables, linear regression analysis was used to assess correlations. For survival analysis, overall survival time (defined as the time between date of operation and death from recurrent disease) was used as follow-up parameter. Patients dying from causes unrelated to breast cancer were censored at the time of death. Kaplan–Meier curves were plotted, and differences between the curves were analysed using the log-rank test. Multivariate analysis was performed with the Cox regression model (enter and remove limits 0.1) to evaluate additional prognostic value of the AI to other prognostic variables. All these tests were carried out with SPSS. *P*-values below 0.05 were regarded as significant.

### RESULTS

The AI showed a non-normal distribution with a range from 1 to 96 and a median value of 10 apoptotic cells per $mm^2$. The AI showed a positive correlation to the MAI in linear regression analysis ($r$ = 0.36, $P < 0.0001$). As shown in Table 1, the AI was not associated lymph node status ($P = 0.08$). Tumour size showed a weak correlation to the AI with large tumours showing a higher AI than small tumours ($P = 0.049$). AI was significantly higher for the ductal and medullary tumour types compared to the tubular, invasive cribriform, mucinous and lobular tumour types ($P = 0.003$). When comparing histologic grade and AI, higher histologic grade was associated with high number of apoptosis ($P < 0.0001$).

**Table 1** Correlations between lymph node status, tumour size, histological type and grade and the apoptotic index

| Variable | | n | Apoptotic index Mean | Median | Range | P-value |
|---|---|---|---|---|---|---|
| Lymph node status | Negative | 86 | 12.5 | 9 | 68 (1–69) | 0.081 |
| | Positive | 86 | 16.1 | 12 | 94 (2–96) | |
| Tumour size | < 2.5 cm | 80 | 12.2 | 9 | 94 (2–96) | 0.049 |
| | ≥ 2.5 cm | 92 | 16.2 | 12 | 68 (1–69) | |
| Histologic type | Ductal, medullary | 132 | 16.0 | 12 | 95 (1–96) | 0.003 |
| | Others | 40 | 8.9 | 8 | 39 (2–41) | |
| Histologic grade | I | 73 | 10.2 | 8 | 48 (1–49) | < 0.0001 |
| | II | 61 | 14.4 | 12 | 92 (4–96) | |
| | III | 38 | 22.2 | 8 | 65 (4–69) | |

**Table 2** Univariate survival analysis results for the total group of patients

| Variable | Cut-off point | n | % survival | P-value | Log-rank |
|---|---|---|---|---|---|
| Tumour size | < 2.5 cm | 80 | 85 | < 0.0001 | 22.3 |
| | ≥ 2.5 cm | 92 | 48 | | |
| Histologic type | Ductal, medullary | 132 | 66 | NS | 0.4 |
| | Others | 40 | 65 | | |
| Histologic grade | I | 73 | 81 | 0.0002 | 16.6 |
| | II | 61 | 57 | | |
| | III | 38 | 49 | | |
| Lymph node status | Negative | 86 | 77 | 0.001 | 10.7 |
| | Positive | 86 | 55 | | |
| MAI | < 10 | 91 | 81 | < 0.0001 | 18.7 |
| | ≥ 10 | 81 | 50 | | |
| Apoptotic index | < 10 | 80 | 78 | 0.0007 | 11.6 |
| | ≥ 10 | 92 | 55 | | |

NS = not significant.

Table 2 shows the results of the univariate survival analysis for the different variables in the total group of patients. Patients with high AI showed shorter overall survival than patients with low apoptotic counts (log-rank 11.6, $P = 0.0007$, Figure 2). As shown in Table 3, the AI was also significant in the subgroup of lymph

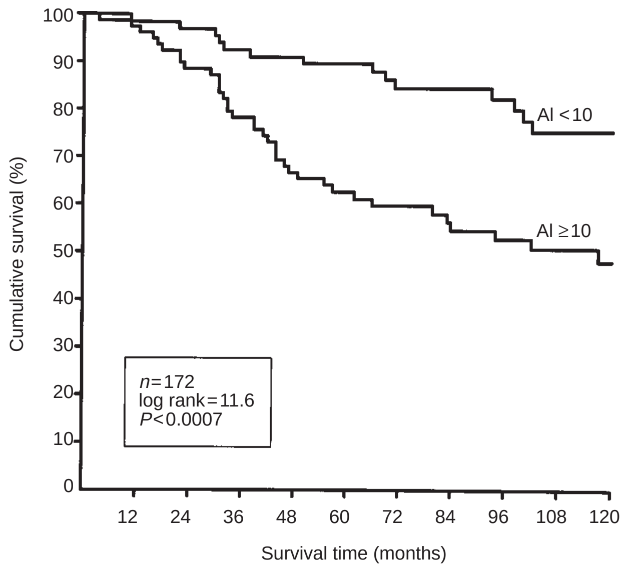

**Figure 2** Survival curves of stage I and II invasive breast cancer patients with low (< 10) and high (≥ 10) AI

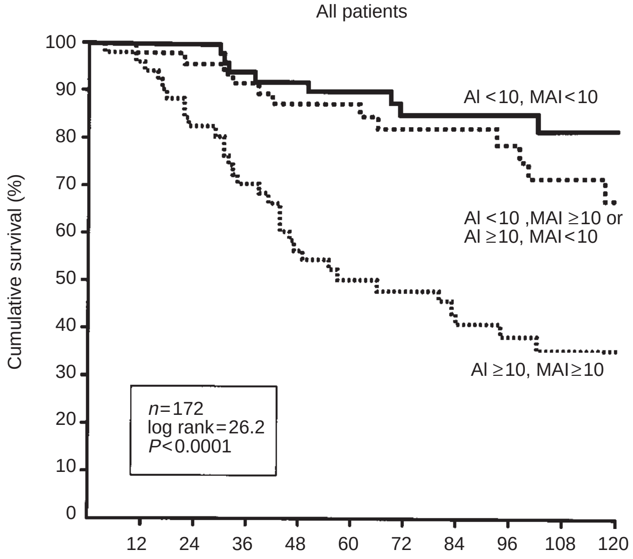

**Figure 3** Survival curves of stage I and II invasive breast cancer patients with low AI and low MAI ($n = 58$, straight line), low AI and high MAI or high AI and low MAI ($n = 55$, dashed line), or high AI and high MAI ($n = 59$, dotted line)

© 2000 Cancer Research Campaign

**Table 3** Univariate survival analysis results for the subgroup of lymph node-positive patients

| Variable | Cut-off point | *n* | % survival | *P*-value | Log-rank |
|---|---|---|---|---|---|
| Tumour size | < 2.5 cm | 33 | 76 | 0.002 | 9.8 |
| | ≥ 2.5 cm | 53 | 42 | | |
| Histologic type | Ductal, medullary | 70 | 57 | NS | 0.0 |
| | Others | 16 | 44 | | |
| Histologic grade | I | 28 | 71 | 0.02 | 8.0 |
| | II | 39 | 51 | | |
| | III | 19 | 35 | | |
| MAI | < 10 | 42 | 74 | 0.003 | 9.2 |
| | ≥ 10 | 44 | 37 | | |
| Apoptotic index | < 10 | 35 | 74 | 0.005 | 8.1 |
| | ≥ 10 | 51 | 41 | | |

NS = not significant.

**Table 4** Univariate survival analysis results for the subgroup of lymph node-negative patients

| Variable | Cut-off point | *n* | % survival | *P*-value | Log-rank |
|---|---|---|---|---|---|
| Tumour size | < 2.5 cm | 47 | 90 | 0.003 | 9.0 |
| | ≥ 2.5 cm | 39 | 55 | | |
| Histologic type | Ductal, medullary | 62 | 77 | NS | 0.2 |
| | Others | 24 | 78 | | |
| Histologic grade | I | 45 | 86 | 0.03 | 6.9 |
| | II | 22 | 67 | | |
| | III | 19 | 62 | | |
| MAI | < 10 | 49 | 86 | 0.005 | 8.0 |
| | ≥ 10 | 37 | 64 | | |
| Apoptotic index | < 10 | 45 | 82 | NS | 1.8 |
| | ≥ 10 | 41 | 72 | | |

NS = not significant.

**Table 5** Multivariate survival analysis results

| Variable | s.e.m. | *P*-value | Exp(B) |
|---|---|---|---|
| Tumour size | 0.0621 | 0.0001 | 1.27 |
| MAI | 0.0057 | 0.001 | 1.02 |
| Lymph node status | 0.3113 | 0.016 | 0.47 |
| Apoptotic index | 0.0078 | 0.030 | 1.02 |

s.e.m. = standard error of the mean, Exp(B) = exponent B.

node positive patients (log-rank 8.1, $P = 0.005$) but not in lymph node-negative patients (Table 4). Tumour size, MAI, lymph node status and AI were shown to be independent prognostic indicators in multivariate analysis as shown in Table 5. Figure 3 shows the survival plot for the combination of AI and MAI. Patients with low AI and low MAI showed the best overall survival whereas patients with high AI and high MAI showed the shortest overall survival (log-rank 26.2, $P < 0.0001$). In the subgroup of lymph node-negative patients, the AI had no additional value to the MAI. In the lymph node-positive group, patients with high number of apoptotic cells and a high MAI showed a significantly shorter overall survival than the patients with either a low AI or a low MAI (log-rank 15.7, $P = 0.0004$).

To compare the number of apoptotic cells with the number of mitotic figures in the same tumour, we converted the MAI to the number of mitotic figures per $mm^2$. Eighty-six per cent of tumours showed a higher number of apoptoses than mitoses per $mm^2$.

### DISCUSSION

In accordance with previous studies, also in the present study, the number of apoptotic cells and the number of mitotic figures showed a strong correlation (Allan et al, 1991; Lipponen et al, 1994; van Slooten et al, 1997; Berardo et al, 1998, Zhang 1998). The close relation between high AI and high MAI suggests common genetic regulators. The c-*myc* oncogene may be one of the genes involved in these processes. c-*myc* can promote cell proliferation in the presence of growth factors. However, c-*myc* induces apoptosis when insulin-like growth factor and platelet-derived growth factor are not available (Evan et al, 1992, 1996). Evan et al (1996) proposed that when c-*myc* is active, there is simultaneous induction of cell proliferation and apoptosis. Amplification of c-*myc* is associated with high proliferation rates and a poor prognosis in invasive breast cancer (Berns et al, 1992; Borg et al, 1992). Besides c-*myc*, other regulators of the cell cycle are known to play a dual role in the regulation of apoptosis and proliferation. Cyclin D1 can bind to cyclin-dependent kinases (cdks) and subsequently phosphorylate retinoblastoma protein (pRb), thereby releasing the E2F transcription factor, thus acting as a promoter of cell proliferation. However, neurons in the developing nervous system undergo apoptosis under the regulation of cyclin D1 (Kranenburg et al, 1996). In a previous study we found no correlation between the number of apoptotic cells and the expression of cyclin D1 in invasive breast cancer (de Jong et al, 1998). Cyclin D1 overexpression was shown to be associated with

© 2000 Cancer Research Campaign a low number of mitotic figures and low proliferation rate in invasive breast cancer (van Diest et al, 1997). Recent reports showed a dual role for growth factors and their receptors in induction of proliferation and apoptosis. The epidermal growth factor receptor (EGFR) when stimulated with a low dose of EGF can prevent A431 cells from undergoing programmed cell death. However, when stimulated with a high dose of EGF, EGF induces inhibition of cell proliferation and induction of apoptosis (Gulli et al, 1996). EGF and its receptor may therefore provide a linked role since increased tyrosine kinase activity can drive two coupled functions, proliferation and programmed cell death.

We found a high AI to be related to a large tumour size. This is in line with the study of Zhang et al (1998), but two other studies found no relation between tumour size and AI (Lipponen et al, 1994; Berardo et al, 1998). A high AI was found in ductal and medullary tumour types compared to a low AI in the more differentiated tumour types. This is in line with the study of Lipponen et al (1994). However, the study of Mustonen et al (1997) found no difference in AI between lobular or ductal breast cancer. As in all previous published studies the AI in grade 3 tumours was about twice as high as in grade 1 tumours and grade 2 tumours show AI in between these two values (Lipponen et al, 1994; Mustonen et al, 1997; van Slooten et al, 1998; Zhang et al, 1998). These results indicate that poorly differentiated, high-grade tumours with a high proliferation rate show a high rate of apoptosis. However, the net rate of proliferation is still so high that they can produce large tumours as indicated by the relation between AI and tumour size.

The role of p53 in mediating proliferation and apoptosis in invasive breast cancer is still not clear. Wild-type p53 expression can lead to G1 arrest, thereby inhibiting proliferation. On the other hand, wt p53 is able to up-regulate bax expression and down-regulate bcl-2 expression thereby stimulating apoptosis. In invasive breast cancer, a large proportion of tumours show strong immunohistochemical p53 staining, which is mostly due to a mutation induced increased half-life of the protein. m-p53 is unable to induce bax expression and thereby loses its ability to induce apoptosis. In ovarian cancer cells, p53 mutation induces resistance to cisplatin as a consequence of loss of the ability of m-p53 to transactivate *bax* (Perego et al, 1996). The breast cancer cell line BT474 which has m-p53 function expresses a high bcl-2/bax ratio and shows resistance to topoisomerase inhibitors treatment (Davis et al, 1998), underlining the inability of m-p53 to down-regulate bcl-2 expression and up-regulate bax expression. This study also showed an association between bcl-2 expression and resistance to apoptosis in breast cancer cells. In a prospective study on the effect of adjuvant therapy in metastatic breast cancer, bax expression was correlated to good treatment response (Sjöström et al, 1998). These studies underline the potential importance of p53, bcl-2 and bax for response to adjuvant chemotherapy. In some patients from our study the number of apoptotic cells seems to be higher than the number of mitotic figures per $mm^2$, suggesting a negative growth rate. This paradox can, however, be explained by the persistence of apoptotic bodies for 30 min to maybe several hours, in contrast to the relatively rapid completion of mitosis (Staunton and Gaffney, 1998). The actual number of proliferating cells in these tumours is therefore probably higher than the number of apoptotic cells, leading to a more plausible positive balance for the net growth of the tumour. Besides, the length of the mitotic phase may be highly variable, especially in DNA aneuploid tumours which makes comparison of the number of apoptotic cells and the number of mitotic figures more difficult until reliable assessment of the duration of the apoptotic and mitotic phases becomes possible.

The AI was shown to be of strong prognostic value in this study. Patients with high AI showed short overall survival compared to patients with low AI. This is in line with a previous study of Lipponen et al (1994) who also found high apoptotic counts to be related to short overall survival. However, Berardo et al, who used a TUNEL assay to detect apoptotic cells in a series of 979 lymph node-positive breast cancer patients, found no correlation to disease-free survival or overall survival when dividing the patients into low or high rate of apoptosis. When patients were divided into four separate groups based on the percentage of apoptotic cells, there was a trend towards worse survival as levels of apoptosis increased (Berardo et al, 1998). However, the TUNEL technique can be low in sensitivity and specificity for staining of apoptotic cells. Some apoptotic cells are not stained while necrotic cells and inflammatory cells may be falsely positively stained. Besides, the methodology for scoring the number of apoptotic cells highlighted by the TUNEL technique is less developed than the 'H&E' technique and requires additional studies. In multivariate analysis we found the AI to be an independent prognostic indicator with also additional prognostic value of the MAI. Anti-apoptotic proteins like bcl-2 and bcl-$x_L$ have been related to low rates of cell death and a favourable prognosis (Sierra et al, 1998; van Slooten et al, 1998) while the pro-apoptotic bax protein showed no relation to the number of apoptotic cells or prognosis in invasive breast cancer in most studies (Sierra et al, 1998; van Slooten et al, 1998; Veronese et al, 1998). The number of apoptotic cells may therefore reflect the net result of the pro- and anti-apoptotic stimuli, providing an easy to use tool to assess the capability of tumours to undergo programmed cell death. Since counting of apoptotic bodies can be performed with good reproducibility (Schepop et al, 1996), it is a promising novel prognostic indicator in invasive breast cancer with additional prognostic value to tumour size, lymph node status and mitotic index. The AI deserves to be studied prospectively, especially with regard to response to adjuvant therapy.

### ACKNOWLEDGEMENTS

We thank Dr Hans Peterse from the Dutch Cancer Institute for providing breast material in an earlier phase. Supported in part by grants #28–2015 and #28–1814 of the Praeventiefonds.

### REFERENCES

Allan DJ, Howell A, Roberts SA, Williams GT, Watson RJ, Coyde JD, Clarke RB, Laidlaw IJ and Potten CS (1991) Reduction in apoptosis relative to mitosis in histologically normal epithelium accompanies fibrocystic change and carcinoma of the premenopausal human breast. *J Pathol* **163**: 337–342

Baak JPA, Dop H van, Kurver PHJ and Hermans J (1985) The value of morphometry to classic prognosticators in breast cancer. *Cancer* **56**: 374–382

Barbareschi M, Caffo O, Veronese S, Leek RD, Fina P, Fox S, Bonzanini M, Girlando S, Morelli L, Eccher C, Pezzella F, Doglioni C, Dalla Palma P and Harris A (1996) Bcl-2 and p53 expression in node-negative breast carcinoma: a study with long-term follow-up. *Hum Pathol* **27**: 1149–1155

Bargou RC, Dainel PT, Mapara MY, Bommert K, Wagener C, Killinich B, Royer HD and Dörken B (1995) Expression of the bcl-2 gene family in normal and malignant breast tissue: low bax-alpha expression in tumor cells correlates with resistance towards apoptosis. *Int J Cancer* **60**: 854–859

Berardo MD, Elledge RM, de Moor C, Clark GM, Osborne CK and Allred DC (1998) Bcl-2 and apoptosis in lymph node positive carcinoma. *Cancer* **82**: 1296–1302

 © 2000 Cancer Research Campaign

Berns EMJJ, Klijn JGM, Putten WLJ van, Staveren IL van, Portengen H and Foekens JA (1992) c-myc amplification is a better prognostic factor than HER2/neu amplification in primary breast cancer. *Cancer Res* **52**: 1107–1113

Borg Å, Baldetorp B, Fernö M, Olsson H and Sigurdsson H (1992) c-*myc* amplification is an independent prognostic factor in postmenopausal breast cancer. *Int J Cancer* **51**: 687–691

Clayton F (1991) Pathologic correlates of survival in 378 lymph node-negative infiltrating ductal breast carcinomas. Mitotic count is the best single predictor. *Cancer* **68**: 1309–1317

Davis PL, Shaiu WL, Scott GL, Iglehart JD, Hsieh TS and Marks JR (1998) Complex response of breast epithelial cell lines to topoisomerase inhibitors. *Anticancer Res* **18**: 2918–2932

Diest PJ van and Baak JPA (1992) The morphometric multivariate prognostic index (MPI) is the strongest prognosticator in premenopausal lymph node negative and lymph node positive breast cancer patients. *Hum Pathol* **22**: 479–489

Diest PJ van, Michalides RJAM, Jannink I, Valk P van der, Peterse HL, Jong JS de, Meijer CJLM and Baak JPA (1997) Cyclin D1 overexpression in breast cancer. Correlations and prognostic value. *Am J Pathol* **150**: 705–711

Doglioni C, Dei Tos AP, Laurino L, Chiarelli C, Barbareschi M and Viale G (1994) The prevalence of Bcl-2 immunoreactivity in breast carcinomas and its clinicopathological correlations, with particular reference to estrogen receptor status. *Virchows Arch* **424**: 47–51

Elston CW (1987) Grading of invasive carcinoma of the breast. In: Page DL, Anderson TJ, eds. Diagnostic histopathology of the breast. Edinburgh: Churchill Livingstone, 300–311

Evan GI, Wyllie A and Gilbert C (1992) Induction of apoptosis in fibroblasts by c-myc protein. *Cell* **69**: 119–128

Evan GI, Harrington E, McCarthy N, Gilbert C, Benedict MA and Nunez G (1996) Integrated control of cell proliferation and apoptosis by oncogenes. In: *Apoptosis and Cell Cycle Control in Cancer*, Thomas NSB (ed), pp. 109–129. BIOS Scientific: Oxford

Farrow SN and Brown R (1996) New members of the Bcl-2 family and their protein partners. *Curr Opinion Genet Develop* **6**: 45–49

Gulli LF, Palmer KC, Chen YQ and Reddy KB (1996) Epidermal growth factor-induced apoptosis in A431 cells can be reversed by reducing the tyrosine kinase activity. *Cell Grow Diff* **7**: 173–178

Heatley MK (1995) Association between the apoptotic index and established prognostic parameters in endometrial adenocarcinoma. *Histopathology* **27**: 469–472

Jannink I, Diest PJ van and Baak JPA (1995) Comparison of the prognostic value of four methods to assess mitotic activity in 186 invasive breast cancer patients: classical and random mitotic activity assessments with correction for volume percentage of epithelium. *Hum Pathol* **26**: 1086–1092

Jong JS de, Diest PJ van, Michalides RJAM, Valk P van der, Meijer CJLM and Baak JPA (1998) Correlation of cyclin D1 and Rb gene expression with apoptosis in invasive breast cancer. *Mol Pathol* **51**: 30–34

Kapucuoglu N, Losi L and Eusebi V (1997) Immunohistochemical localization of Bcl-2 and Bax proteins in in situ and invasive breast carcinomas. *Virchows Arch* **430**: 17–22

Krajewski S, Krajewska M, Shabaik A, Miyashita T, Wang HG and Reed JC (1994) Immunohistochemical determination of in vivo distribution of Bax, a dominant inhibitor of Bcl-2. *Am J Pathol* **145**: 1323–1336

Krajewski S, Blomqvist C, Franssila K, Krajewska M, Wasenius VM, Niskanen E and Reed JC (1995) Reduced expression of the proapoptotic gene BAX is associated with poor response rates to combined chemotherapy and shortened survival in women with metastatic breast adenocarcinoma. *Cancer Res* **55**: 4471–4478

Kranenburg O, Eb AJ van der and Zantema A (1996) Cyclin D1 is an essential mediator of apoptotic neuronal cell death. *EMBO J* **15**: 46–54

Leoncini L, Del Vecchio MT and Magha T (1993) Correlations between apoptotic and proliferation indices in malignant non-Hodgkin's lymphoma. *Am J Pathol* **142**: 755–763

Linden JC van der, Baak JPA, Lindeman J, Hermans J and Meijer CJLM (1987) Prospective evaluation of prognostic value of morphometry in patients with primary breast cancer. *J Clin Pathol* **40**: 302–306

Lipponen P (1999) Apoptosis in breast cancer: relationship with other pathological parameters. *Endocrine-Rel Cancer* **6**: 13–16

Lipponen P and Aaltomaa S (1994) Apoptosis in bladder cancer as related to standard prognostic factors and prognosis. *J Pathol* **173**: 333–339

Lipponen P, Collan Y and Eskelinen MJ (1991) Volume corrected mitotic index (M/V index), mitotic activity index (MAI), and histological grading in breast cancer. *Int Surg* **76**: 245–249

Lipponen P, Aaltomaa S, Kosma VM and Syrjanen K (1994) Apoptosis in breast cancer as related to histopathological characteristics and prognosis. *Eur J Cancer* **30A**: 2068–2073

Minn AJ, Boise LH and Thompson CB (1996) Bcl-$x_S$ antagonizes the protective effects of Bcl-$x_L$. *J Biol Chem* **271**: 6306–6312

Miyashita T, Kitada S, Krajewski S, Horne WA, Delia D and Reed JC (1995) Overexpression of Bcl-2 protein increases the half-life of p21 (Bax). *J Biol Chem* **270**: 26049–26052

Mustonene M, Taunio H, Pääkkö P and Soini Y (1997) The extent of apoptosis is inversely associated with bcl-2 expression in premalignant and malignant breast lesions. *Histopathology* **31**: 347–353

Perego P, Giarola M, Righetti SC, Supino R, Caserini C, Delia D, Pierotti MA, Miyashita T, Reed JC and Zunino F (1996) Association between cisplatin resistance and mutation of *p53* gene and reduced bax expression in ovarian carcinoma cell systems. *Cancer Res* **56**: 556–562

Schepop HAM, Jong JS de, Diest PJ van and Baak JPA (1996) Counting of apoptotic cells: a methodological study in invasive breast cancer. *Mol Pathol* **49**: M214–M217

Sierra A, Castellsagué X, Coll T, Mañas S, Escobedo A, Moreno A and Fabra A (1998) Expression of death-related genes and their relationship to loss of apoptosis in $T_1$ ductal breast carcinomas. *Int J Cancer* **79**: 103–110

Sjöström J, Krajewski S, Franssila K, Niskanen E, Wasenius VM, Nordling S, Reed JC and Blomqvist C (1998) A multivariate analysis of tumour biological factors predicting response to cytotoxic treatment in advanced breast cancer. *Br J Cancer* **78**: 812–815

Slooten HJ van, Vijver MJ van de, Velde CJ van de and Dierendonck JH van (1998) Loss of Bcl-2 in invasive breast cancer is associated with high rates of cell death, but also with increased proliferation activity. *Br J Cancer* **77**: 789–796

Staunton MJ and Gaffney EF (1998) Apoptosis: basic concepts and potential significance in human cancer. *Arch Pathol Lab Med* **122**: 310–319

Uyterlinde AM, Schipper NW, Baak JPA, Peterse H and Matze P (1988) Limited prognostic value of cellular DNA content to classical and morphological parameters in invasive ductal breast cancer. *Am J Clin Pathol* **89**: 301–307

Veronese S, Mauri FA, Caffo O, Scaioli M, Aldovini D, Perrone G, Galligioni E, Doglioni C, Dalla Palma P and Barbareschi M (1998) Bax immunohistochemical expression in breast carcinoma: a study with long term follow-up. *Int J Cancer* **79**: 13–18

Weisman JH, Jonker RR, Keijzer R, Velde CJH van de, Cornelisse CJ and Dierendonck JH van (1993) A new method to detect apoptosis in paraffin sections: in situ end-labelling of fragmented DNA. *J Histochem Cytochem* **41**: 7–12

Yang E and Korsmeyer SJ (1996) Molecular thanatopsis: a discourse on the Bcl-2 family and cell death. *Blood* **88**: 386–401

Zhang GJ, Kimijima I, Abe R, Watanabe T, Kanno W, Kiyoshi H and Tsuchiya A (1998) Apoptotic index correlates to bcl-2 and p53 protein expression, histological grade and prognosis in invasive breast cancers. *Anticancer Res* **18**: 1989–1998

*© 2000 Cancer Research Campaign* 
